# Back to Basic: Do Children with Autism Spontaneously Look at Screen Displaying a Face or an Object?

**DOI:** 10.1155/2013/835247

**Published:** 2013-12-08

**Authors:** Marie Guimard-Brunault, Nadia Hernandez, Laetitia Roché, Sylvie Roux, Catherine Barthélémy, Joëlle Martineau, Frédérique Bonnet-Brilhault

**Affiliations:** ^1^CHRU de Tours, Centre Universitaire de Pédopsychiatrie, 2 Boulevard Tonnellé, 37044 Tours Cedex 9, France; ^2^Université François Rabelais de Tours, 60 rue du Plat D'Etain, 37020 Tours Cedex 1, France; ^3^UMR Inserm U 930, Équipe 1: Imagerie et Cerveau, Université François Rabelais de Tours, Tours, France; ^4^UMR Inserm U 930, Équipe 1: Imagerie et Cerveau, CHRU de Tours-Hôpital Bretonneau, 2 boulevard Tonnellé, Bât B1A, 1er Etage, 37044 Tours Cedex 9, France

## Abstract

Eye-tracking studies on exploration of faces and objects in autism provided important knowledge but only in a constraint condition (chin rest, total time looking at screen not reported), without studying potential differences between subjects with autism spectrum disorder (ASD) and controls in spontaneous visual attention toward a screen presenting these stimuli. This study used eye tracking to compare spontaneous visual attention to a screen displaying a face or an object between children with autism and controls in a nonconstraint condition and to investigate the relationship with clinical characteristics in autism group. Time exploring screen was measured during passive viewing of static images of faces or objects. Autistic behaviors were assessed by the CARS and the BSE-R in autism group. In autism group, time exploring face screen and time exploring object screen were lower than in controls and were not correlated with degree of distractibility. There was no interaction between group and type of image on time spent exploring screen. Only time exploring face screen was correlated with autism severity and gaze impairment. Results highlight particularities of spontaneous visual attention toward a screen displaying faces or objects in autism, which should be taken into account in future eye-tracking studies on face exploration.

## 1. Introduction

Autism spectrum disorder (ASD) represents a significant public health concern and their prevalence is 60–70 per 10000 [1]. ASD is a developmental disorder characterized by impairments in communication and social interaction and by repetitive and stereotyped behavior and restricted interests [2].

Some authors suggest that social interaction impairment is linked to face processing impairment [3–5]. Face processing is one of the most studied areas of visual processing in ASD [6], and eye-tracking studies have made a large contribution to research in this field. Many eye-tracking studies focused on exploration strategies of faces in ASD. A consistent finding in eye-tracking studies is less visual exploration of the eyes by individuals with ASD compared to typically developing individuals [7–12], associated in some cases to greater exploration of the mouth [9–11].

Some other eye-tracking studies investigated both visual attention to faces and objects in ASD and control groups [13–15]. While the two first authors found no group differences in visual attention to faces versus objects [13, 14], Rice et al. reported a significantly reduced fixation on faces in ASD compared to controls [15].

Another major contribution of eye-tracking studies is that they allow studying associations between visual attention to both face and objects and the clinical characteristics of ASD [10, 14–16]. Klin et al. indeed found a significant correlation between fixation on mouths and objects and social functioning: increased focus on mouths predicted better social adjustment and less autistic social impairment, whereas more time on objects predicted the opposite [10]. In addition, Rice et al. found an association between higher fixation on the inanimate environment and greater social disability [15]. A recent study found that face processing skills were significantly correlated with measures of attention to faces [14]. In another clinical domain, Sasson et al. found that, within their autism group, overall severity of repetitive behavior symptoms correlated positively with exploration of object pictures and negatively with perseveration on social pictures [16].

Although these eye-tracking studies provide important knowledge about visual attention to faces and objects in autism, these results are subject to a potential bias that might limit the generalization of these findings and explain mixed results. These studies only assessed visual attention to stimuli in a constraint condition. Indeed, they either used a chin rest to ensure that children necessarily look at screen and/or only calculated time exploring stimuli by dividing the time spent looking at stimuli by the total amount of time looking at screen without reporting this latter measure. By focusing on exploration of faces and objects stimuli when subjects looked at screen, they do not take into account the potential difference between children with ASD and control subjects in terms of spontaneously visual attention toward a screen presenting faces or objects stimuli. In a nonconstraint condition, Martineau et al. have already highlighted a difference between ASD and controls in terms of pupil responses during visual scanning of face and object stimuli [17]. What about spontaneous visual attention to a screen displaying a face or an object in autism?

This study aimed to compare spontaneous visual attention to a screen displaying a face or an object between children with ASD and controls using an eye-tracking approach in a nonconstraint condition. We also aimed to study in autism group the link between spontaneous visual attention to a screen displaying a face or an object and clinical characteristics. We hypothesized that children with ASD would spend less time exploring screen than controls for both stimuli (face and object). We further hypothesized that there would be no interaction between group and type of image—face versus object—on time spent exploring the screen. Finally, we hypothesized that spontaneous visual attention to a screen displaying a face or an object would be inversely correlated to autism severity but not linked to age or distractibility.

## 2. Method

### 2.1. Participants

Seventy-two subjects—24 with autism and 48 controls—aged 3 to 16 years old participated in this study.

Children with ASD were recruited from the Department of Child Psychiatry, University Hospital Center of Tours, France. The diagnosis of autism was based on the clinical judgment of an expert based on the criteria of the Diagnostic and Statistical Manual of Mental Disorders, Fourth Edition (DSM-IV-TR) [2]. This assessment was conducted in a tertiary medical center expert in autism which uses the Autism Diagnostic Interview-Revised (ADI-R) [18] and/or the Autism Diagnostic Observation Schedule (ADOS) [19]. All children with ASD met criteria for autistic disorder.

Typical subjects were recruited from the local community. The exclusion criteria were a history of developmental disabilities, psychiatric, or neurological disorders. They all had a normal history of cognitive development and were progressing in age-appropriate education programs. Typical and autism groups did not differ in age (M age autism group = 9.53, SD = 3.71; M age typical group = 9.22, SD = 3.37; *F*_1,70_ = 0.12; *P* = .73).

For all participants, inclusion criteria consisted of normal or corrected vision ≥10/10 and no history of any eye disorder. None of the participants were taking any prescribed or over-the-counter medication. Written informed consent was obtained from all participants' parents. This study conformed to the Declaration of Helsinki.

### 2.2. Materials

#### 2.2.1. Stimuli

**Figure 1:**
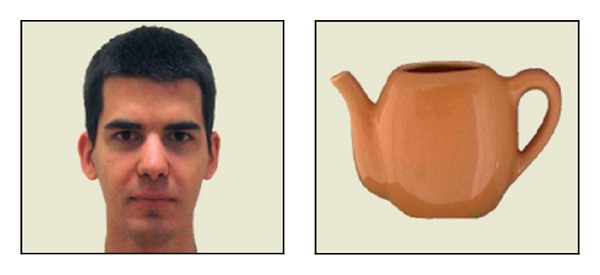
The two types of visual stimuli: neutral faces and objects.

Visual stimuli included 10 neutral faces—photographs of humans aged 18–35 years on a beige background—and 10 objects from daily life on a beige background (Figure 1). These stimuli have been previously used and validated elsewhere [8]. They were presented in color and matched on image size and luminosity.

#### 2.2.2. Eye-Tracking System

We recorded gaze direction with an eye-tracking system described in detail elsewhere [8] including a computer equipped with two cameras which film the eyes of a subject looking at pictures presented on the computer screen. The software supporting the camera system captured cornean-illumination through infrared diodes, thus eliminating constraints on the subject, who remained free to move. The FaceLAB monitoring system determined eye direction, and Gaze Tracker software provided measurement and real-time analysis of the duration of exploration of the entire screen. The eye-tracking system recorded the eye position every 0.017 s. The size of the stimuli was 37.7 cm × 30 cm with a resolution of 1024 × 768 px (visual angle = 27°). We studied total time tracked defined as time spent on the screen during the stimulus projection.

### 2.3. Procedure

**Figure 2:**
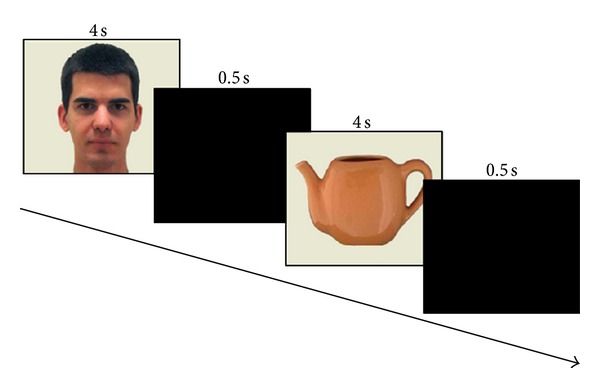
Procedure.

Each of the 20 visual stimuli was successively projected on a 21 in. computer screen for 4 s with an interstimulus interval of 0.5 s consisting of a blank black slide (Figure 2). Gaze was spontaneously centered in the middle of the screen between each stimulus with this blank black slide. Central position of gaze was systematically checked by the experimenter for each patient and for each blank black slide during the experiment using the control screen of the gaze tracker system. The order of presentation of the stimuli was randomized between subjects. We wanted to study spontaneous visual attention towards stimuli, so there was no instruction before or during the experiment. Participants sat in a comfortable armchair, 90 cm from the computer screen, with no chin rest and in the darkness. After a brief calibration, gaze direction was recorded by the eye-tracking system during the entire session of visual stimuli projection.

### 2.4. Measures

#### 2.4.1. Eye-Tracking Measures

For both groups (autism and control), the eye-tracking system measured the time spent exploring the entire screen in both “face screen” and “object screen” conditions. In both groups, the average time exploring the entire screen did not differ between the 10 stimuli in the same condition. There was no effect of the order of the stimuli. To achieve our statistical analyses, we therefore averaged the results of the 10 stimuli of each condition for each subject. We thus used two measures called “time exploring face screen” and “time exploring object screen.” These two measures were the mean duration per each 4 s exposure time for each condition. We did not exclude any participant for these two measures.

#### 2.4.2. Clinical Measures

Several clinical measures for the autism group were obtained during multidisciplinary assessment. All clinical measures were based on clinical observations conducted by clinicians expert in autism. Full-scale intelligence quotient (IQ) was estimated using reliable and appropriate scales. For each child, we chose the intelligence measure appropriate to mental age: the Brunet Lézine-R, a psychomotor developmental test for infants 1–30 months of age (Brunet and Lézine 1976, the French version of the Gesell scale, revised form 2001) [20], the EDEI-R (Echelles différentielles d'efficiences intellectuelles, Perron-Borelli 1978, revised form 1996), a cognitive test for children 30 months–9 years of age [21], and the Wechsler Intelligence Scale for Children, Third Edition (WISC-III), a cognitive test for children between the ages of 6 and 16 inclusive [22].

Children with ASD were also assessed with the Childhood Autism Rating Scale (CARS) [23] designed to assess the severity of autistic syndrome and with the Behavior Summarized Evaluation-Revised (BSE-R) [24] developed for the quantitative assessment of autistic behavior (Table 1). In particular, we studied Factor 1 of BSE-R “Interaction disorder” (13 items: aloneness, ignores people, poor social interaction, abnormal eye contact, does not make an effort to communicate using voice and words, lack of appropriate facial expression and gestures, lack of initiative, poor activity, inappropriate relating to inanimate objects or to dolls, stereotyped sensorimotor activity, unstable attention, easily distracted, bizarre responses to auditory stimuli, does not imitate the gestures or voices of others, and does not share emotion) reflecting relational defect and quantifying the severity of autistic behavior and BSE-R item scores reflecting functions associated with visual attention (item 4: abnormal eye contact, item 23: unstable attention, easily distracted). Previous studies already pulled items of BSE-R out of the scale to study correlations between neurophysiological variables and target autistic behaviors [25, 26].

### 2.5. Data Analysis

We used Statistica 6.1 software (StatSoft, Inc., Tulsa, OK). We used repeated measures ANOVA to study the effects of group and type of image on eye tracking measures. Before these tests, we checked for the homogeneity of variances with a Levene test. In autism group, Spearman correlations were used to assess the relationship between visual patterns and clinical variables. *P* value ≤.05 was considered significant for all statistical analyses. All analyses were two tailed.

## 3. Results

### 3.1. Clinical Assessments

Results for the autism group with the various scales assessed during multidisciplinary assessment are reported in Table 2.

### 3.2. Time Exploring Screen

#### 3.2.1. Descriptive Statistics

The mean time exploring face screen was 1.28 s (SD = .91) for children with ASD and 3.04 s (SD = .77) for controls. The mean time exploring object screen was 1.13 s (SD = .67) for children with ASD and 2.85 s (SD = .91) for controls.

#### 3.2.2. Autism Group versus Controls

**Figure 3:**
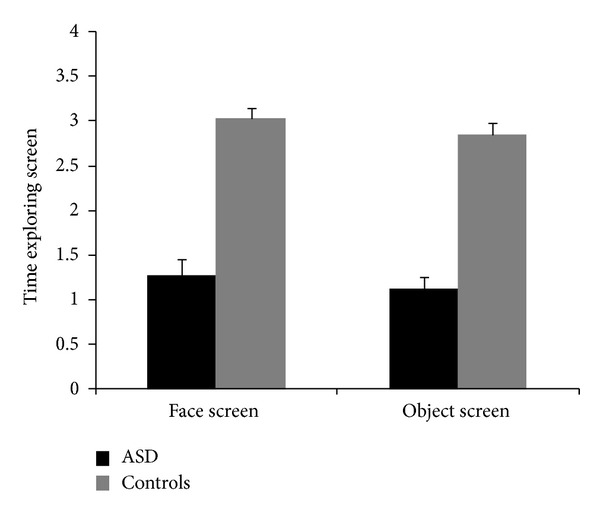
Time exploring face screen and time exploring object screen in children with ASD and controls.

The ANOVA showed a significant group effect on time exploring the screen (*F*_1,70_ = 81.66; *P* < .0001) and a significant effect of the type of image (*F*_1,70_ = 5.00; *P* = .03); their interaction was not significant (*F*_1,70_ = 0.06; n.s.). Children with ASD spent less time than controls exploring screen whatever the type of image. All participants spent more time exploring face screen than object screen (Figure 3).

#### 3.2.3. Clinical Correlates in Autism Group

Correlation tests between clinical variables and time exploring face or object screen are described in Table 3.

**Figure 4:**
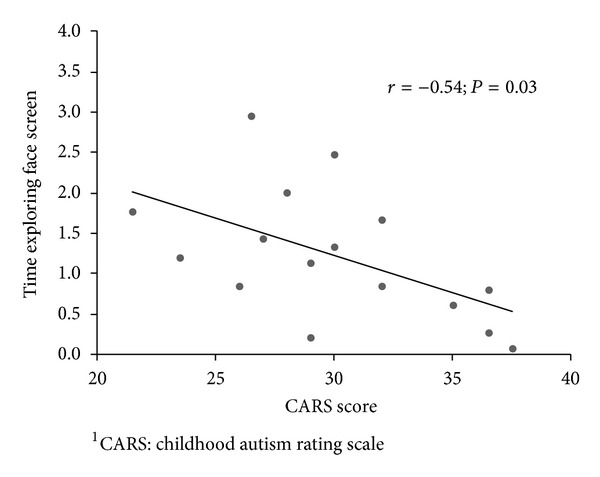
Correlation between time exploring face screen and CARS score^1^ in autism group.

In the autism group, there was no significant correlation between the time exploring face screen and chronological age, full-scale IQ, item 23 of BSE-R (distractibility), or factor 1 score of BSE-R. There was a significant negative correlation between the time exploring face screen and CARS score: the higher the score CARS was, the less time was spent exploring face screen (Figure 4). This correlation remained significant after adjustment with full-scale IQ (Figure 4). We also found a significant negative correlation between the time exploring face screen and score for item 4 of BSE-R (abnormal eye contact): the more impaired the gaze, the less time was spent exploring face screen. There was no significant correlation between the time exploring object screen and any of the clinical variables (chronological age, full-scale IQ, score CARS, item 23 of BSE-R (distractibility), factor 1 score of BSE-R, or item 4 of BSE-R (abnormal eye contact).

#### 3.2.4. Maturation Effect on Time Exploring Screen

In controls, there was a correlation between chronological age and the time exploring face screen (*n* = 48; *r* = .32; *P* = .02): as age increased, the time exploring face screen increased. There was no correlation between chronological age and time exploring object screen (*n* = 48; *r* = .25; n.s.). In the autism group, there was no correlation between chronological age and the time exploring face screen (*n* = 24; *r* = .15; n.s.) nor between chronological age and the time exploring object screen (*n* = 24; *r* = .06; n.s.).

## 4. Discussion

In this study, we report that children with ASD spent significantly less time than controls exploring a screen presenting a face or an object. In addition, we found no interaction between group and type of image—face versus object—on the time spent exploring screen. The low screen exploration by the autism group was not associated with the degree of distractibility. Finally, the time exploring face screen was specifically associated with autism severity and gaze impairment; this was not the case for the time exploring object screen.

First, supporting our original hypothesis, children with ASD spent significantly less time than controls spontaneously exploring the screen, regardless of whether the screen showed a face or an object. Two main hypotheses can be drawn to explain this low spontaneous visual attention in autism: deficits in visual attention and enhanced perception processing strategies [27–38]. Deficits in visual attention have been reported in autism, including “subselecting” important stimuli and/or hyperselecting irrelevant stimuli [27–29], a low attention engagement [30, 31], a disengagement and visual attention shift disorder [32–34], and abnormal visual orientation skills [32, 34, 35]. The use of more specific attention tests would help explore the various components of visual attention potentially involved in this low spontaneous visual attention in autism. The hypothesis of enhanced perceptual function in autism [36–38] could also explain why children with ASD spent less time exploring the screen displaying stimuli: they might capture the information provided by the stimuli more quickly.

Consistent with our prediction, there was no interaction between group and type of image—face versus object—on time spent exploring the screen. This finding is in line with previous studies [13, 14] which were conducted using a constraint condition. This may reflect a global alteration of spontaneous visual attention in autism [39–41], which would not be specific to visual attention toward faces stimuli.

Our final hypothesis was that spontaneous visual attention to a screen displaying a face or an object would be inversely correlated to autism severity but not linked to age or distractibility. We partly confirmed this final hypothesis. First, we confirmed that the time exploring a screen (projecting a face or an object) was not related to the degree of distractibility, as assessed by item 23 of BSE-R. Second, we found no association between time exploring screen for both stimuli and the severity of developmental delay. However, this result should be tempered by the lack of IQ assessment in controls needed to further explore this hypothesis. The low screen exploration in ASD compared to controls could be explained by a difference in cognitive abilities. Third, we found no association between age and time exploring screen for both stimuli in autism group. We found developmental maturation of the time exploring face screen in controls, consistent with previous descriptions of the development of expertise in face processing during typical development [42]. There was no evidence of such developmental maturation of the time exploring face screen in the autism group. Indeed, we did not find any correlation between chronological age and time exploring face screen in the autism group. Neither the autism group nor controls showed developmental maturation for the time exploring object screen. These results are in line with particularities of visual attention to faces, differing between children with ASD and controls, not present for visual attention to objects. Finally, in the autism group, the time exploring face screen was inversely correlated with the CARS score and was therefore related to the severity of autistic behaviors. This study is the first demonstration, as far as we are aware, of a significant association between time spent exploring screen toward faces and autism severity in a nonconstraint condition. However, the time exploring face screen was not correlated with the Factor 1 score of the BSE-R. The differences between these two scales may explain this result. Careful analysis of the details of the various scale items indicates that the CARS is more focused than the BSE-R on eyes, attention to others, and visual perception. Moreover, item 4 of BSE-R score, specifically related to abnormal eye contact, was inversely correlated with time exploring face screen: the more impaired the gaze of children with ASD, the shorter time spent exploring the screen with a face. To refine the results and the specificity of these behavioral patterns, we studied the time exploring object screen. In contrast to our predictions, for such images of objects, we did not find the same clinical correlations. Time exploring object screen was not correlated with any of the clinical variables that were tested or with the severity of autistic behaviors. This result highlights specific peculiarities in spontaneous visual attention toward a screen displaying a face compared to spontaneous visual attention toward a screen displaying an object in autism.

This study has some limitations. A larger sample would have allowed more detailed study of each age and an improvement in statistical power, especially for correlations. It would also have been interesting to assess the subjects using attention tests to better investigate the involvement of visual attention abnormalities in the atypical processing of faces in autism. As another limitation of our study, the screen contained only 1 image surrounded by a beige background. This approach could be considered as a potential constraint, and it would be interesting to propose simultaneously several images on the screen with the same methodology. Such study could assess ocular preference between several images by determining first whether subjects do or do not spontaneously look at the screen. Our results should also be tempered by the lack of IQ assessment in controls, which could be a potential confounding factor that might explain low exploration in ASD, since most of them had low IQ. Finally, we used photographs of faces, such that they were not animated, and they were not in any sort of natural environment; this may have influenced our findings [43].

In conclusion, this eye-tracking study demonstrates a lower spontaneous visual attention towards both a screen displaying an object and a screen displaying a face in autism. In autism group, spontaneous visual attention for both stimuli was not associated with age and degree of distractibility. Only spontaneous visual attention to face screen was associated with autism severity. The mechanisms underlying this specificity need to be investigated.

Our study has research and practical implications. As for research implication, we confirmed our hypothesis of a difference between children with ASD and control subjects in terms of spontaneously visual attention towards a screen presenting faces or objects stimuli. As far as we are aware, this study is the first to demonstrate this difference. Previous eye-tracking studies on face and object exploration did not take into account this difference since they either used a chin rest to ensure that children necessarily look at screen, and/or only calculated time exploring stimuli by dividing the time spent looking at stimuli by the total amount of time looking at screen without reporting this latter measure. Our study highlights the need to take into account this difference in the interpretation of eye-tracking studies in face and object exploration in autism, by stating whether the investigators used a constraint or a nonconstraint condition and by reporting the total amount of time spontaneously looking at screen in addition to previously used measures. Another implication of this work is a clinical one. Indeed, it would be interesting to apply this nonconstraint protocol during treatment. Treatment followup with the BSE-R may help to characterize the development of social and nonsocial behavior, including spontaneous visual attention to faces and objects, and this could be used to adjust the treatment plan and thereby improves the social prognosis of children with ASD.

## Figures and Tables

**Table 1:** Items of BSE-R scale.

Items of BSE-R scale	
(1) Aloneness	
(2) Ignores people	
(3) Poor social interaction	
(4) Abnormal eye contact	
(5) Does not make an effort to communicate using voice and words	
(6) Lack of appropriate facial expression and gestures	
(7) Stereotyped vocal and verbal utterances, echolalia	
(8) Lack of initiative, poor activity	
(9) Inappropriate relating to inanimate objects or to doll	
(10) Irresistible and/or ritual use of objects	
(11) Intolerance of change and to frustration	
(12) Stereotyped sensorimotor activity	
(13) Agitation, restlessness	
(14) Bizarre posture and gait	
(15) Autoaggressiveness	
(16) Heteroaggressiveness	
(17) Mild anxiety signs	
(18) Mood difficulties	
(19) Disturbance of feeding behavior	
(20) Does not try to be clean (stools or urine), plays with stools	
(21) Individual bodily activities	
(22) Sleep problems	
(23) Unstable attention, easily distracted	
(24) Bizarre responses to auditory stimuli	
(25) Variability	
(26) Does not imitate the gestures or voices of others	
(27) Child too floppy, lifeless	
(28) Does not share emotion	
(29) Paradoxical sensitivity to touching and contact	

**Table 2:** Clinical assessments in autism group.

	*N*	Mean ± SD	Range
Full IQ scale^1^	24	63.3 ± 20.1	19–99
CARS score^2^	16	30.0 ± 4.7	21.5–37.5
Factor 1 of BSE-R^3^	18	29.7 ± 10.7	16–46
Item 4 of BSE-R^3^	18	2.3 ± 1.2	1–5
Item 23 of BSE-R^3^	18	3.1 ± 1.1	1–5

^1^IQ: intelligence quotient.

^2^CARS: Childhood Autism Rating Scale.

^3^BSE-R: Behavior Summarized Evaluation-Revised.

**Table 3:** Clinical correlates in autism group.

	Time exploring face screen			Time exploring object screen		
	*n*	*r*	*P*	*n*	*r*	*P*
Chronological age	24	0.15	n.s.	24	0.06	n.s.
Full IQ^1^ scale	24	0.19	n.s.	24	0.20	n.s.
CARS score^2^	16	−0.54	0.03	16	−0.31	n.s.
CARS score*	16	−0.54	0.03	16		
Factor 1 of BSE-R^3^	18	−0.32	n.s.	18	−0.13	n.s.
Item 4 of BSE-R^3^	18	−0.47	0.047	18	−0.16	n.s.
Item 23 of BSE-R^3^	18	−0.28	n.s.	18	0.03	n.s.

^1^IQ: intelligence quotient.

^2^CARS: Childhood Autism Rating Scale.

^3^BSE-R: Behavior Summarized Evaluation-Revised.

*Partial correlation adjusted for full IQ scale.

n.s.: nonsignificant.
